# Evidence of an active role of dreaming in emotional memory processing shows that we dream to forget

**DOI:** 10.1038/s41598-024-58170-z

**Published:** 2024-04-15

**Authors:** Jing Zhang, Andres Pena, Nicole Delano, Negin Sattari, Alessandra E. Shuster, Fiona C. Baker, Katharine Simon, Sara C. Mednick

**Affiliations:** 1 University of California, Irvine, USA; 2 SRI International, Menlo Park, USA

**Keywords:** Human behaviour, Forgetting

## Abstract

Dreaming is a universal human behavior that has inspired searches for meaning across many disciplines including art, psychology, religion, and politics, yet its function remains poorly understood. Given the suggested role of sleep in emotional memory processing, we investigated whether reported overnight dreaming and dream content are associated with sleep-dependent changes in emotional memory and reactivity, and whether dreaming plays an active or passive role. Participants completed an emotional picture task before and after a full night of sleep and they recorded the presence and content of their dreams upon waking in the morning. The results replicated the emotional memory trade-off (negative images maintained at the cost of neutral memories), but only in those who reported dreaming (Dream-Recallers), and not in Non-Dream-Recallers. Results also replicated sleep-dependent reductions in emotional reactivity, but only in Dream-Recallers, not in Non-Dream-Recallers. Additionally, the more positive the dream report, the more positive the next-day emotional reactivity is compared to the night before. These findings implicate an active role for dreaming in overnight emotional memory processing and suggest a mechanistic framework whereby dreaming may enhance salient emotional experiences via the forgetting of less relevant information.

## Introduction

The function of dreams has been a topic of fascination and speculation throughout human history, with evidence of dream-related beliefs and practices dating back to ancient civilizations. In traditional Chinese culture, for example, dreams were seen as a portal to the future. *The Duke of Zhou’s Interpretation of Dreams* served as a reference for ancient Chinese people to use dreams to guide their future behaviors, which remains popular to this day. In ancient Egyptian society, dreams were often recorded and interpreted by priests, believing that gods offered guidance and prophesy to individuals through dreams. One of the most influential modern perspectives on the function of dreams was developed by Sigmund Freud. In his book *The Interpretation of Dreams*, Freud argued that dreams are the expression of unconscious desires and repressed thoughts, and they serve as a form of psychological release allowing us to process and resolve unconscious conflicts. Though compelling, Freud’s dream theory did not put forth testable hypotheses that would help determine the mechanistic value of dreams for human functioning. Since that time, the study of dreaming has become a scientific discipline. Pivotal studies in the 1960s on the function of dreaming employed meticulous dream diaries and sleep-interruption paradigm to reveal a nuanced relationship between waking experiences and dream content^1^, laying groundwork for recent studies to understand the neural basis^2^ and function of dreaming^3,4^.

Due to the emotional nature of dream content^5^, contemporary hypotheses focus on how dreams affect emotional processing of waking experiences. A prominent debate exists regarding the passivity or activity of dreaming on wake, with proponents of the passive role theory arguing that dreams are a mere reflection of waking affect^6–8^, and proponents of the active role theory proposing that thoughts and feelings are transformed during dreams^9–12^. A prominent proponent of the passive role, the continuity theory of dreaming, hypothesizes that the emotional temperature of dreams will be positively associated with that of waking experiences^7,8,13,14^. According to this theory, the content and affect of dreams mirror our daytime experiences and thoughts. For example, if we have a stressful day at work, we may have unpleasant dreams about work-related tasks that night and have similar feelings of stress the following day. This hypothesis is supported by studies that report a continuity between self-rated emotions before, during, and after dreaming^15,16^. Barbeau and colleagues reported that pre-sleep negative emotions were associated with negative dream emotions and negative morning emotions, suggesting dream mood is consistent with waking affect. Similarly, another study found that dream mood and content were positively associated with mood the next morning^17^. Additionally, Sikka and colleagues tracked dreaming and next-day emotional reactivity for five days, and they reported that the more negative the dreams, the more negative the next-morning affect^8^.

Conversely, the emotion regulation theory of dreaming proposes dreams play an active role in reprocessing and regulating waking affect^9,11,12,18^. According to this theory, dreams provide a safe space to experience and process emotions, particularly negative ones. The emotion regulation theory of dreaming is different from the continuity theory of dreaming as it proposes that dreams lead to a functional change in emotion regulation during waking. For example, a person who is anxious about giving a speech may have an emotionally charged dream related to this experience due to the anxiety they felt throughout the day, and the next day they may report feeling less anxious. In one seminal study by Cartwright and colleagues, depressed divorcees slept in the lab and were awakened four times during rapid eye movement (REM) sleep to report their dreams. A year later, the participants’ depression symptoms were assessed again through surveys. Researchers found that depressed divorcees who dreamed about their ex-spouses were more likely to have a significant reduction in depressive symptoms at 1-year follow up^11^. A replication study reported that depressed divorcees whose dreams were more detailed with memories and emotions tend to have decreased depressive symptoms a year later compared to those with less detailed dreams^9^. In another study, participants were experimentally stressed before sleep; those who dreamed about the stressful event had a more positive attitude towards the experiment the next morning than those who did not dream about it, suggesting that dreaming helps transform emotional reactivity and process difficult, waking experiences^19^. In support of this idea, a recent study showed that participants with a high prevalence of fear-related dreams had decreased activation in fear-related brain areas during wakefulness, suggesting dreaming might benefit the emotion regulation process^20^.

The simulation theory of dreaming also emphasizes the active role of dreaming in emotional processing, positing that dreams serve to simulate threats and rehearse coping methods in a virtual context^21–23^. Several studies have provided support for this theory by showing that engagement in pre-sleep rehearsal of real-life concerns and problems may manifest this waking content during dreams^24,25^. Similarly, Hartmann argued that dreaming about a stressful situation helps the dreamer connect this memory to other related memories and prepares the dreamer for the future^26,27^. It is worth noting that even though both the emotion regulation theory and simulation theory assume an active role of dreaming in the emotion process, they lead to differing predictions. The emotion regulation theory argues that the downregulation of affect during dreams reflects an adaptive emotion regulation function, while the simulation theory predicts that dreams serve to simulate stressful or threating situations that does not necessarily lead to adaptive responses in next-day wakefulness. For example, nightmares indicate a failure in emotion regulation as per the emotion regulation theory, but they serve as a functional threat simulation system according to the simulation theory.

One way to disentangle the key difference between the active and passive theories of dreaming, i.e., whether dreams support the transformation of emotional reactivity, is to adopt an experimental paradigm from the sleep-dependent memory consolidation field, which investigates how memory performance changes pre-to-post by an intervening sleep period. By correlating natural variations in the quality of sleep with performance, researchers can identify candidate mechanisms for consolidation processes. To this end, researchers can investigate changes in overnight emotional reactivity in people who report dreaming compared with people who do not report dreaming. Previous studies have examined the association between dreaming and next-day affect, but not how affect might change pre-to-post sleep^8,15,17^. The current study aims to answer this question by measuring overnight changes in emotional reactivity to negative and neutral pictures in those who report dreaming (Dream-Recallers) and those who do not recall dreaming (Non-Dream-Recallers).

Sleep-dependent memory studies have demonstrated a role for REM sleep in preferentially preserving emotional memories at the cost of neutral memories that were encoded at the same time, an effect termed the emotional trade-off effect^28,29^. REM sleep is characterized by vivid dreams, rapid eye movements, muscle atonia, theta oscillatory activity (4–7 Hz), and is accompanied by increased acetylcholine and decreased serotonin and norepinephrine levels^30,31^. Moreover, the sleep-to-forget, sleep-to-remember (SFSR) hypothesis posited that emotional experiences have separable memory and emotional components that get processed differently during sleep, with the memory component strengthened by theta oscillation coordinated reactivation of encoding-related regions during REM sleep, while the affective tone is reduced by decreased aminergic activities^32^.

Physiological markers of REM sleep associated with a reduction in reactivity to emotional stimuli have also been found, with theta^33^ and gamma power^34^, as well as minutes in REM^35^. However, the effect of REM sleep on emotional reactivity yields mixed results. While some studies show that REM sleep facilitates emotion regulation^36,37^, other studies suggest that REM sleep might exacerbate emotional arousal^38^. For example, one study reported that participants showed decreased arousal ratings to negative stimuli after REM sleep deprivation^38^. Interestingly, this study explored the role of dream mood in this process and found that dreams rated as less negative lead to decreased valence ratings to negative stimuli the next morning^38^. Despite dreams being reported in all sleep stages, REM sleep is characterized by the most frequent recall of vivid dreams and the incorporation of recent memory sources^14,39,40^. Thus, dreaming may be an overlooked area to explain the inconsistent findings on how REM sleep affects emotional reactivity. Moreover, prior studies have linked dreaming with sleep-dependent memory consolidation^14,39,41–43^. Wamsley and colleagues also reported that dreaming about a spatial navigation task during a nap^44^ and a night of sleep^45^ boosted post-sleep performance. Taken together, dreaming might play a role in both the memory consolidation and the emotional regulation aspects of emotional memory processing.

The current study investigates whether dreaming plays a passive or active role in memory consolidation and emotion regulation by specifically addressing how the presence and content of dreams affect the prioritization of emotional memories over neutral memories^28^, as well as changes in valence and arousal ratings to negative stimuli overnight. Participants performed an emotional picture task before and after a night of sleep and completed a dream report upon waking. We examined how dream recall and content affects performance while controlling for levels of depression, anxiety, and emotion regulation, as these factors play a role in dream recall and dream content^46–48^.

## Methods

### Participants

One hundred and twenty-five healthy female adults (*M*age = 33.69 ± 12.07 years) with no history of neurological, psychological, sleep disorders, or other chronic illnesses were recruited for the study. Only female participants were recruited for this study as the data is taken from a larger study focusing on the effects of menstrual cycle on sleep. Fifty of them participated in the experiment in-person at the sleep laboratory, while 75 of them participated remotely due to the COVID-19 pandemic. All participants signed informed consent, which was approved by the Institutional Review Boards at the University of California, Irvine, and all methods were performed in accordance with relevant guidelines/regulations. Exclusion criteria included advanced circadian phase (defined as having a habitual bedtime after 02:00 am and a habitual wake time after 10:00 am), sleep disorder, personal or familial history of diagnosed psychopathology, substance abuse/dependence, loss of consciousness greater than 2 min or a history of epilepsy, current use of psychotropic medications, and any cardiac or respiratory illness that may affect cerebral metabolism, which was determined through self-report. Participants received monetary compensation for their participation (approximately $15/hr).

### Procedure

All participants had an online orientation meeting with trained research personnel to explain the eligibility criteria and have the participant sign the informed consent. Then, participants completed a series of validated surveys on depression (BDI-II, Beck’s Depression Inventory, 2nd Edition)^49^, anxiety (GAD-7, Generalized Anxiety Disorder 7-item)^50^ and emotion regulation (DERS, Difficulties in Emotion Regulation Scale)^51^.

**Figure 1:**
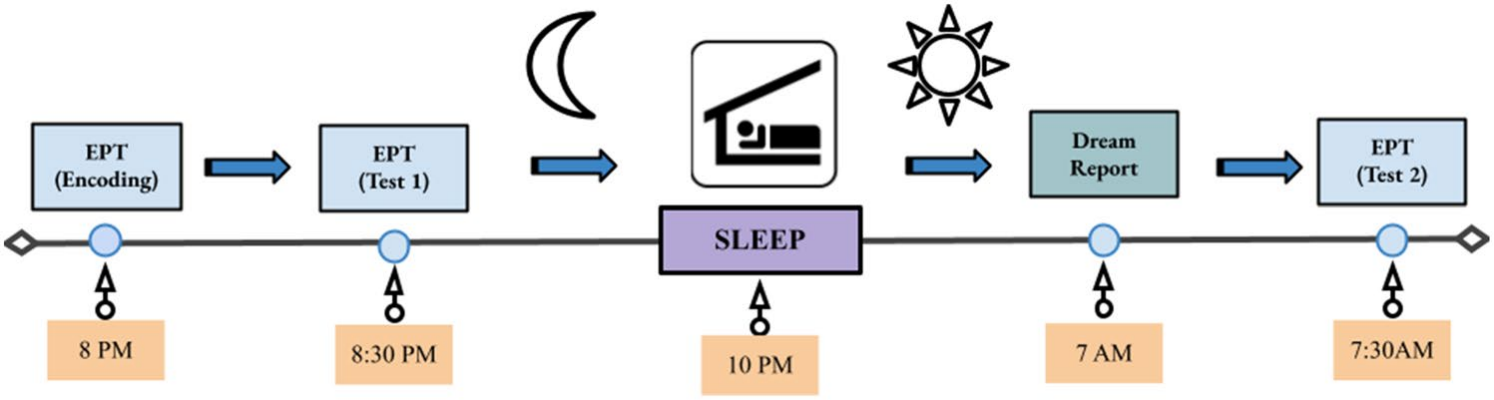
Study Protocol. At 8 PM, participants encoded images from the EPT, emotional picture task, followed by an immediate test. Next, participants slept either at home or in the lab depending on their method of testing being remote or in-person, respectively. Upon awaking, participants reported the presence and description of their dreams and completed a delayed EPT test.

On the day of the experiment, participants reported to the study via Zoom (remote) or to the Sleep and Cognition (SaC) Laboratory (in-person) at around 7:30 pm. They were given an encoding session on the emotional picture task (EPT) at 8:00 pm followed by an immediate test to establish baseline (Test 1). The EPT was administered via an online platform, PsychoPy (Pavlovia.org)^52^, for the remote subjects, while the in-lab participants used MATLAB to complete the task. Then, they slept at home or in a private bedroom at the sleep laboratory. Sleep was monitored by a commercial wearable device the OURA ring. Upon waking in the morning, participants reported their dreaming electronically through an online survey platform (Qualtrics, Provo, UT). The diary assessed whether they recalled having a dream the previous night. If dreaming was recalled, participants reported the details of their dream(s) and rated the mood of their dream(s) on a 7-point Likert scale from extremely negative to extremely positive. After they filled out the sleep diary, participants were tested on the EPT with a trained research personnel over Zoom or in person at the sleep laboratory (Test 2) approximately 2 h after awakening (an overview of the experimental procedure is outlined in Fig. 1).

### OURA ring

Each participant received an Oura ring (Gen 2, firmware version 2.43.1; manufactured by Ōura Health Oy, based in Oulu, Finland). The Oura ring is a wearable device with various sensors, including infrared photoplethysmography (PPG), negative temperature coefficient (NTC), and a 3-D accelerometer. These sensors are located inside the ring and touch the palm side of the finger. The ring is water-resistant and meant to be worn continuously during daily activities. Participants were instructed to wear it on their non-dominant hand's middle, ring, or index finger at all times during the study, except when charging it or engaging in activities that could potentially damage it. Each participant created an anonymized Oura account linked to their study identification number, downloaded the Oura App, and consented to connect their account to the Oura Cloud service. The research team monitored the data collected on the ŌURA cloud daily for each participant to ensure proper data collection. If no data were recorded on any given day, the team followed up with the participant. Studies have shown that the ŌURA ring is highly reliable in detecting sleep–wake patterns, with accuracies, specificities, and sensitivities ranging between 0.88 and 0.89^53^.

### Task

**Figure 2:**
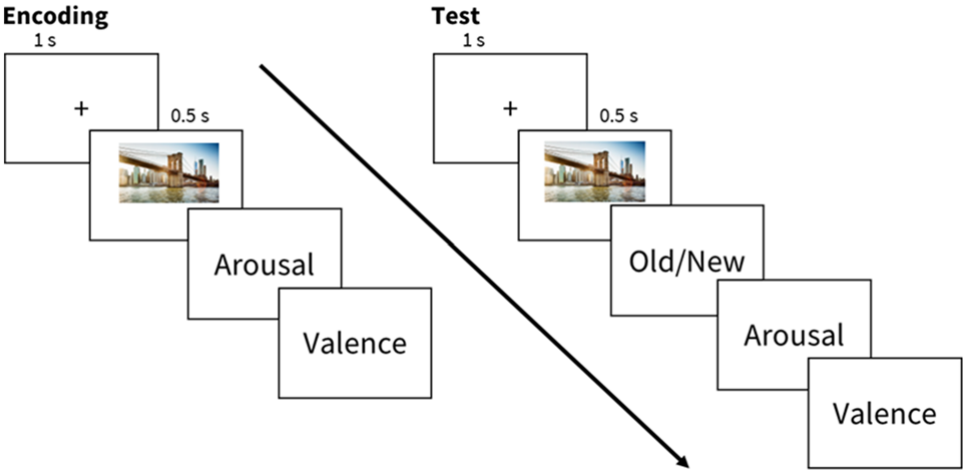
Emotional picture task (EPT) schematic.

The EPT consisted of an encoding phase and two retrieval phases. During encoding, participants viewed 20 negative and 20 neutral pictures for 0.5 s. A fixation marker was presented for 1 s before each picture. Eight additional neutral pictures were used as controls for primacy and recency effects. After viewing each picture, participants were prompted to rate their levels of arousal and valence on a 9-point Likert scale. A score of 1 indicated low arousal (for arousal) or negative feelings (for valence), while a score of 9 indicated high levels. There were two retrieval tests- one immediately following encoding (Test 1) and the second following a night of sleep (Test 2). Each retrieval tested half of the encoded pictures plus 20 novel pictures (10 negative and 10 neutral). After viewing each picture, participants judged if the image was old or new, in addition to their arousal and valence levels. There was no time limit for participants to respond. Participants viewed each picture for 0.5 s and a fixation marker for 1 s in between pictures (Fig. 2).

To measure memory performance, we calculated the discriminability index, d’, using the difference of z-scores for the hit rate (% of pictures correctly identified as present) and the false alarm rate (% of pictures incorrectly identified as present), i.e., d’ = z(hr)–z(fa), at both test 1 and test 2. Then, we calculated the difference scores between test 1 and test 2 (test 2–test 1) to examine performance change overnight. We also calculated the difference in arousal and valence ratings between test 1 and test 2 (test 2–test 1).

### Statistical analysis

We categorized subjects into Dream-Recallers and Non-Dream-Recallers depending on whether they reported having dreamed the previous night. Out of a total of 125 participants, 3 did not provide dream data. Among the 122 participants who submitted dream reports, 51 were classified as Dream-Recallers, reporting having dreams, while 71 were classified as Non-Dream-Recallers, indicating no reported dreams. Among the 51 Dream-Recallers, 21 were in-lab participants and 30 were remote participants. Among the 71 Non-Dream-Recallers, 26 were in-lab participants and 45 were remote participants. Note that for Non-Dream-Recallers, this categorization is unable to distinguish whether a person actually dreamed without recollecting the dream or whether they in fact didn’t dream at all. We conducted independent sample t-tests between Dream-Recallers and Non-Dream-Recallers on OURA sleep measures and EPT measures. We additionally used repeated-measures ANOVA with Dreaming (yes/no) as the between-subject variable and Picture Valence (negative/neutral) as the within-subject variable. We also included the depression, anxiety, and emotion regulation measures as covariates. Finally, we conducted correlations between dream mood ratings and EPT measures, including the memory performance, valence and arousal ratings at each test and overnight change. Within the framework positing an active role of dreams in emotional processing, we anticipated that dream mood ratings would correlate with the overnight changes in EPT measures. If the results were contrary to our expectations, such that dream mood ratings exhibit positive correlations with EPT measures both pre- and post-sleep, while not necessarily correlating with overnight changes, the data would align with the passive model. Given the hypothesis-driven nature of our correlation analysis, we chose not to apply corrections for multiple comparisons.

### Significance statement

This study asks why we dream. Building on prior work demonstrating a link between sleep and the processing of emotional memories, we examine whether dreaming alters overnight memory and emotional reactivity on an emotional picture task. We found that participants who reported dreaming exhibited an emotional memory trade-off, prioritizing retention of negative images over neutral memories, a pattern that was absent in those who did not recall their dreams. Moreover, dreaming was associated with decreased emotional reactivity to negatives memories the following day, with reduced reactivity tied to more positive dream content. We provide the first empirical support for dreaming's active involvement in sleep-dependent emotional memory processing, suggesting that dreaming after an emotional experience might help us feel better in the morning.

## Results

### Comparing remote vs in-lab participants

**Figure 3:**
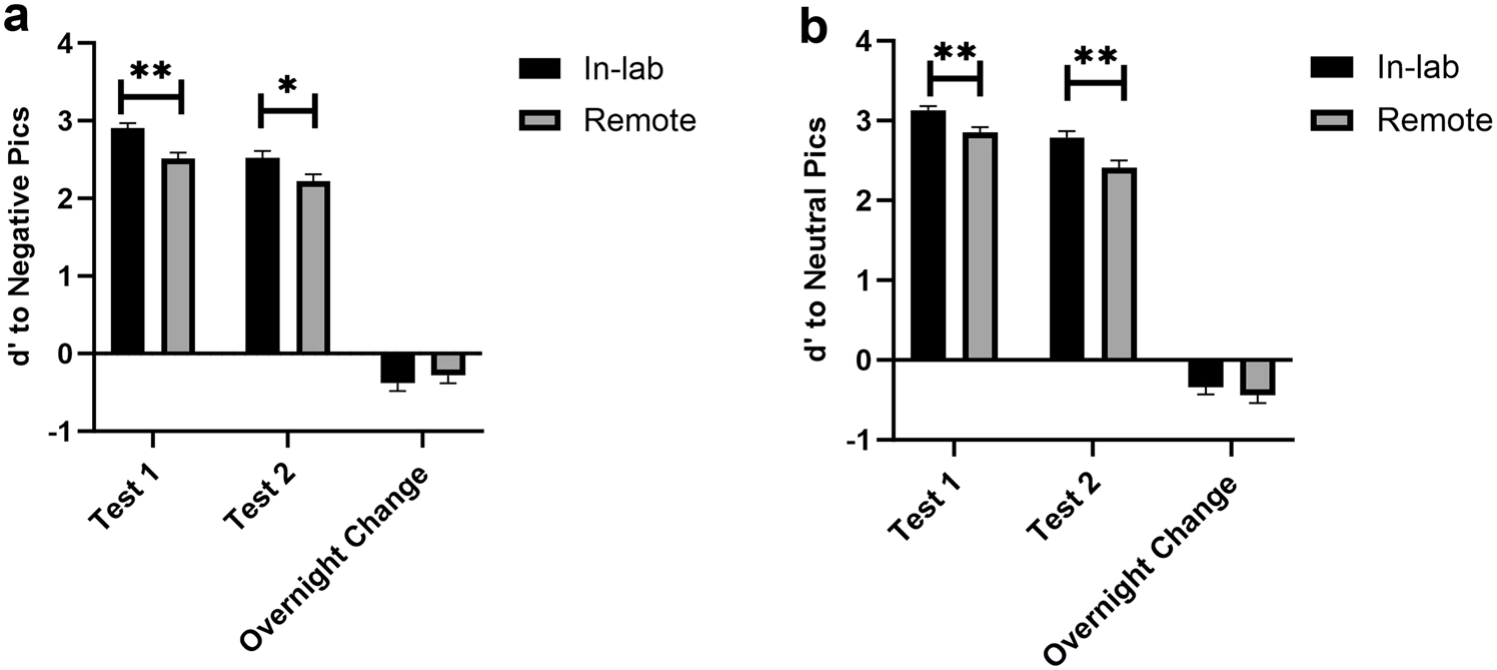
Comparing in-lab and remote participants’ memory performance at Test 1 (immediate), Test 2 (delayed), and overnight change (Test 2–Test 1) for (**a**) negative (Test 1 t_122_ = 3.50, *p* < 0.01; Test 2 t_122_ = 2.17, *p* < 0.03) and (**b**) neutral pictures (Test 1 t_122_ = 2.75, *p* < 0.01; Test 2 t_122_ = 3.11, *p* < 0.01). Despite the significant differences between in-lab versus remote memory performance at Test 1 and Test 2, the overnight change in memory, for both negative and neutral pictures, was not different between in-person and remote participants.

Before combining the data from remote and in-lab participants, we tested cohort differences across key measures, including emotional reactivity, memory performance, sleep, and depression/anxiety. Valence and arousal ratings were not significantly different between the in-lab and remote participants for test 1, test 2, or overnight change. For memory performance, the in-lab participants showed significantly better performance in memory for both negative and neutral images compared to the remote participants at test 1 (negative images: t_122_ = 3.50,* p* < 0.01, neutral images t_122_ = 2.75,* p* < 0.01) and test 2 (negative images: t_122_ = 2.17, *p* < 0.03, neutral images t_122_ = 3.11, *p* < 0.01) (Fig. 3). Notably, the in-lab memory boost may be related to the controlled environment of the sleep laboratory whereas the nature of at-home remote testing may increase the potential for distractions. In addition, the in-lab participants scored higher on the emotion regulation scale compared to the remote participants (t_114_ = 2.92, *p* < 0.01) as measured by DERS, suggesting in-lab participants had more difficulty regulating emotions. There was no significant difference between in-lab and remote participants on BDI-II, GAD-7, and sleep architecture (*p*s > 0.05).

Importantly, the overnight change in memory did not differ between in-lab and remote participants (negative images: t_122_ = − 0.64, *p* = 0.55, neutral images t_122_ = 0.74, *p* = 0.46, Fig. 3), which was our primary metric for investigating the role of dreaming in emotional memory processing. Moreover, even though the in-lab participants scored higher in emotion regulation, the emotion regulation score was not correlated with overnight memory or emotional reactivity changes (Neutral images: *p*_d’_ = 0.76, *p*_valence_ = 0.80, *p*_arousal_ = 0.11; Negative images: *p*_d’_ = 0.80, *p*_valence_ = 0.40 *p*_arousal_ = 0.42) ). Given this lack of significant differences between the cohorts in our measurements of interest, we combined both groups in our subsequent analyses of the overnight difference scores.

### Sleep architecture between dream-recallers and non-dream-recallers

We first examined sleep architecture between Dream-Recallers and Non-Dream-Recallers measured by the wearable device OURA (Table 1). Out of 122 participants with dream reports, 21 had incomplete OURA data. Therefore, we had sleep architecture for 44 Dream-Recallers and 57 Non-Dream-Recallers. The two groups had similar amounts of Light sleep, Deep sleep, REM sleep, Wake and sleep efficiency (*p*s > 0.05). Dream-Recallers had significantly longer total sleep time (TST) compared to Non-Dream-Recallers (t_99_ = − 2.13 *p* = 0.04).

**Table 1:** Sleep architecture. Means ± SD. TST, total sleep time; REM, nonrapid eye movement sleep; SE, sleep efficiency.

Sleep stage	Non-dream recallers	Dream-recallers	*p*-values
TST (min)	452.68 (69.95)	482.25 (67.97)	0.04
Light (min)	205.92 (54.09)	224.92 (51.89)	0.08
Deep (min)	115.14 (42.71)	116.86 (38.63)	0.83
REM (min)	81.36 (36.05)	86.92 (36.96)	0.45
Wake (min)	50.26 (22.94)	53.54 (33.83)	0.56
SE (%)	88.97 (4.34)	89.14 (5.75)	0.87

### Emotional memory trade-off in dream-recallers and non-dream-recallers

Next, we examined how dreaming affects memory consolidation for neutral and negative pictures. We first compared memory performance between the two groups during Test 1 and found no significant difference (*p*s > 0.1) for either negative (Dream-Recallers: M_d’_ = 2.56 ± 0.68; Non-Dream-Recallers: M_d’_ = 2.73 ± 0.60) or neutral images (Dream-Recallers: M_d’_ = 2.91 ± 0.61; Non-Dream-Recallers: M_d’_ = 2.99 ± 0.53). To directly probe the association between dreaming and emotionality of the images, we conducted a repeated measures ANOVA with Dream (Recall/ Non-Recall) as the between-subject factor, Picture Valence (negative/neutral) as the within-subject factor, and mood surveys (GAD-7, BDI-II and DERS) as covariates. Critically, there were no significant differences between Dreamers and Non-Dream-Recallers in the questionnaires examining emotion regulation (DERS: t_98_ = − 0.13, *p* = 0.90), depression (BDI-II: t_98_ = − 0.19, *p* = 0.85) and anxiety (GAD-7: t_98_ = 0.89, *p* = 0.38).

**Figure 4:**
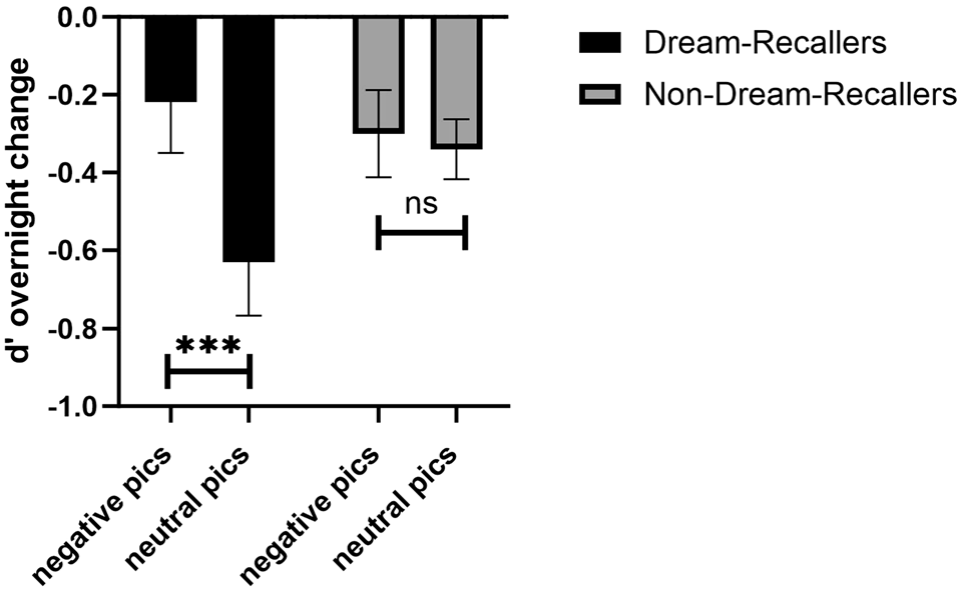
Emotional memory trade-off (d prime in overnight memory change) for negative and neutral pictures for Dream-Recallers and Non-Dream-Recallers. The trade-off effect is shown as negative images maintained at the cost of neutral memories. We found a significant interaction Dream Recalls and Picture Valence (F(1,95) = 4.29, *p* = 0.04). Post hoc tests using Bonferroni suggest Dream-Recallers had less forgetting for negative pictures (M = − 0.22 ± 0.83) compared to neutral (M = − 0.63 ± 0.88) (mean difference = 0.41, t_94_ = 2.96, *p* = 0.02), a pattern not seen in Non-Dream-Recallers (mean difference = 0.04, t_94_ = 0.30, *p* = 1).

The main effect of Picture Valence was not significant (*p* = 0.41), suggesting that participants’ memory for neutral and negative pictures were at similar levels when collapsing across Dream-Recallers and Non-Dream-Recallers. However, when Dreaming was added into the model, we found a significant interaction between the Dream Recall and Picture Valence (F(1,95) = 4.29, *p* = 0.04, Fig. 4). This suggests that dreaming was critical for the sleep-dependent emotional trade-off between maintaining memory for negative pictures at the cost of memory for neutral pictures. Interestingly, the interaction between Dream Recall and Picture Valence was not significant without adding mood surveys as covariates (*p* = 0.23), suggesting that these mood factors modulated the association between dreaming and emotional memory consolidation.

**Figure 5:**
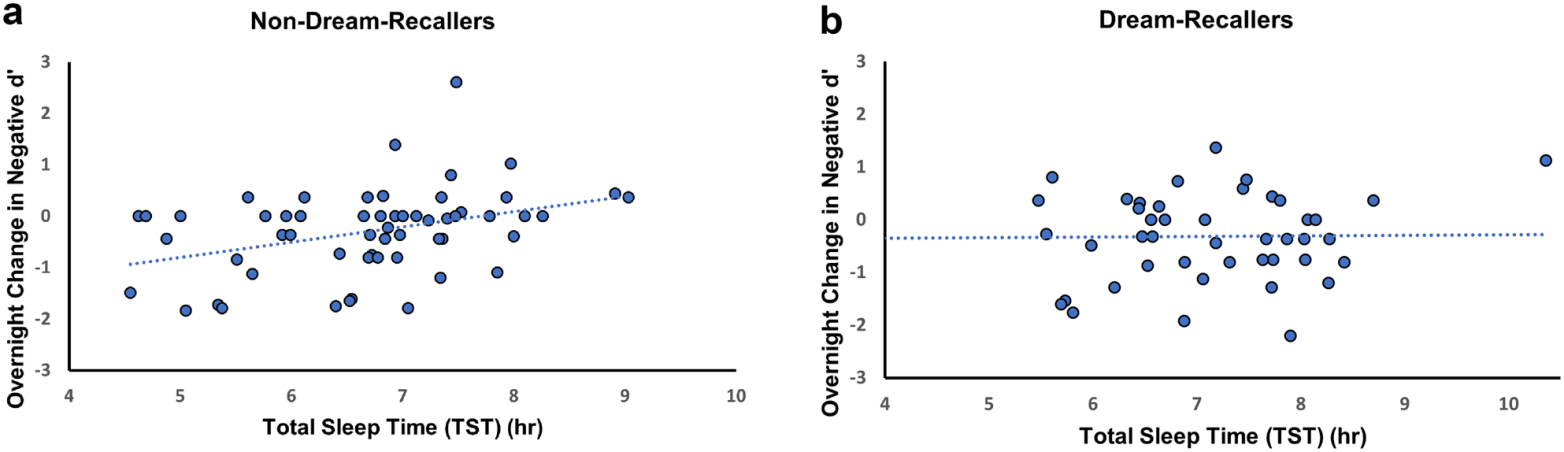
Association between total sleep time (TST) and overnight change in d’ for negative images for (**a**) non-dream-recallers and (**b**) dream-recallers. (**a**) A positive correlation between TST and overnight d’ change for negative pictures indicates longer sleep duration is associated with better overnight memory consolidation for negative images in non-dream-recallers (r = 0.37, *p* = 0.005). (**b**) No significant correlation between TST and overnight d’ change was found for dream-recallers (r = 0.15, *p* = 0.32),

The observed difference in TST between Dream-Recallers and Non-Dream-Recallers could mediate the association between dreaming and memory processing, possibly due to reduced morning homeostatic sleep pressure, lighter morning sleep stages, and increased likelihood of dream recall. To address this potential mediation, we have further explored the role of total sleep time in dreaming and emotional processing. Interestingly, TST was positively associated with overnight change in d’ for negative images in Non-Dream-Recallers (r = 0.37, *p* = 0.005) (Fig. 5a), but not in Dream-Recallers (r = 0.15, *p* = 0.32) (Fig. 5b), suggesting that, independent from dream recall, longer sleep time may enhance retention of emotionally salient information, but not necessarily the forgetting of neutral memories. Valence and arousal ratings for negative and neutral images as well as d’ change for neutral images were not significantly correlated with TST for Dream-Recallers (Neutral images: *p*_d’_ = 0.71, *p*_valence_ = 0.52, *p*_arousal_ = 0.18; Negative images: *p*_valence_ = 0.26, *p*_arousal_ = 0.62) or Non-Dream-Recallers (Neutral images: *p*_d’_ = 0.53, *p*_valence_ = 0.31, *p*_arousal_ = 0.64; Negative images: *p*_valence_ = 0.69, *p*_arousal_ = 0.68). We were curious whether Dream-Recallers had a later waketime, spending more time in REM-rich cycles. However, we did not find a significant difference between Dream-Recallers and Non-Dream-Recallers in waketime (t_96_ = 0.28, *p* = 0.78) or bedtime (t_96_ = 1.18, *p* = 0.24) as measured by OURA. When adding TST to the model examining Dream(Recall/Non-Recall) x Emotional Memory trade-off (negative/neutral) with mood surveys as covariates, the interaction between Dreams Recall and Memory was no longer significant (*p* = 0.21), suggesting that increasing memory for negative images with extended sleep in Non-Dream-Recallers reduced the variance accounted for by dream recall.

The trade-off effect is shown as negative images maintained at the cost of neutral memories. To further investigate the emotional memory trade-off effect in dreaming, we conducted a repeated-measures ANOVA for Dream-Recallers and Non-Dream-Recallers separately, with the d’ change from test 2 to test 1 as the repeated measure (2 levels, negative and neutral), and ratings from DERS, BDI-II and GAD-7 as covariates. We found a significant effect in Dream-Recallers (F(1,37) = 4.47, *p* = 0.04), but not Non-Dream-Recallers (F(1,55) = 0.36, *p* = 0.55) (Fig. 4). Post hoc tests using Bonferroni correction revealed Dream-Recallers had less forgetting for negative pictures (M = − 0.22 ± 0.83) compared to neutral (M = − 0.63 ± 0.88) (mean difference = 0.41, t_94_ = 2.96, *p* = 0.02), but Non-Dream-Recallers did not show this effect (mean difference = 0.04, t_94_ = 0.30, *p* = 1), which indicates that the emotional memory trade-off effect occurred only in those who were able to recall a dream.

### Emotional reactivity in dream-recallers and non-dream-recallers

We turned to the emotional reactivity measure to investigate whether dreaming was specifically associated with decreased overnight reactivity. For all participants, we found a general decrease in emotional reactivity across sleep, with decreased arousal ratings overnight for both negative (t_124_ = 4.02,* p* < 0.001) and neutral pictures (t_124_ = 2.09,* p* < 0.05). The valence rating decreased significantly overnight for negative (t_124_ = 6.94,* p* < 0.001) but not neutral pictures (t_124_ = 0.84,* p* > 0.05).

**Figure 6:**
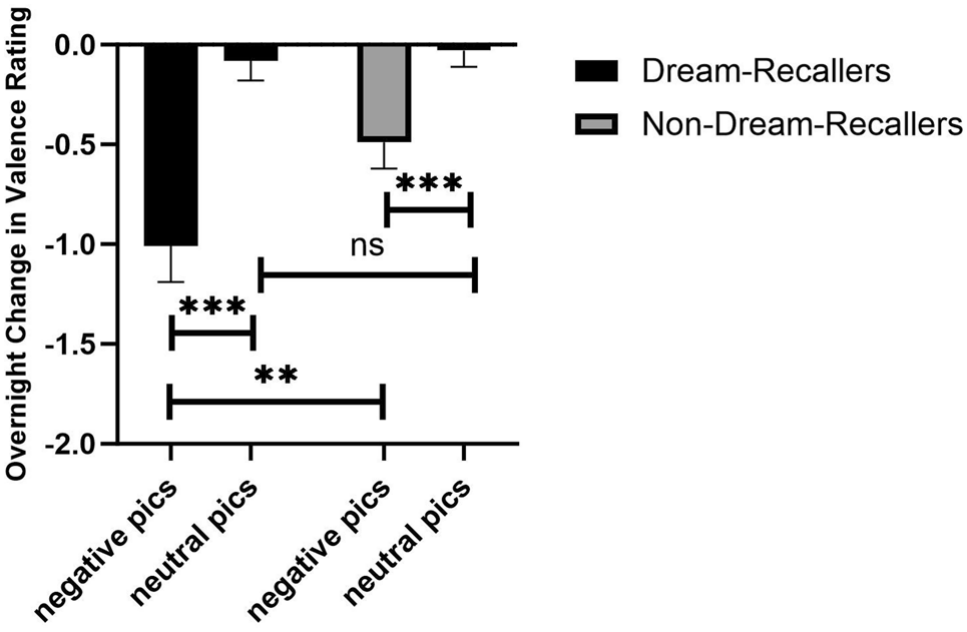
Emotional valence change for negative and neutral pictures for Dream-Recallers and Non-Dream-Recallers. Dream-Recallers showed a significant decrease in valence for negative pictures compared to non-Dream-Recallers (t_120_ = 2.50, *p* = 0.01), but no difference for neutral pictures (t_120_ = 0.43, *p* = 0.67). Both Dream-Recallers (t_50_ = − 4.85, *p* < 0.001) and Non-Dream-Recallers (t_70_ = − 3.52, *p* < 0.001) also showed greater decreases in valence for negative compared to neutral pictures.

**Figure 7:**
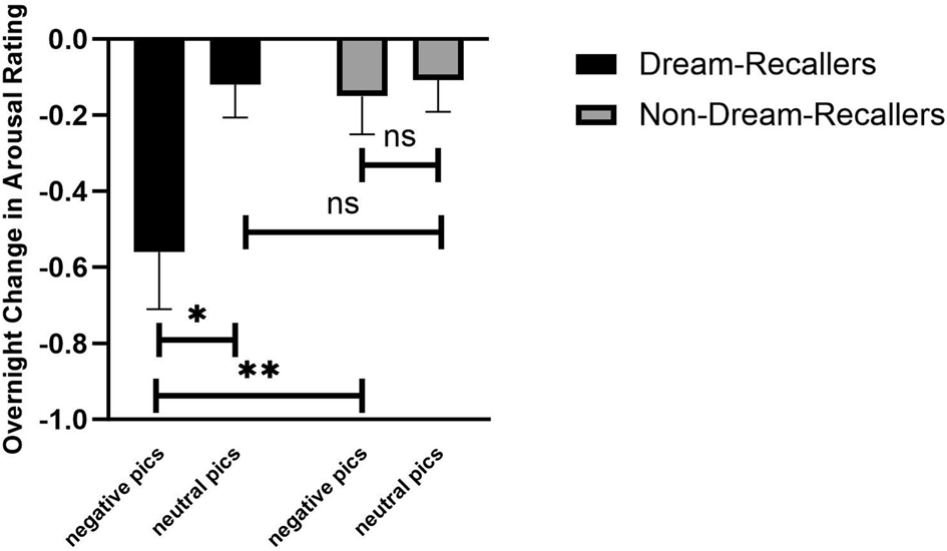
Emotional arousal change for negative and neutral pictures for Dream-Recallers and Non-Dream-Recallers. Dream-Recallers showed a significant decrease in arousal for negative pictures compared to non-Dream-Recallers (t_120_ = 2.40, *p* < 0.02), but no difference for neutral pictures (t_120_ = 0.10, *p* = 0.92). Dream-Recallers also showed a significant decrease in arousal for negative compared to neutral pictures (t_50_ = − 2.91, *p* < 0.005), a pattern no shown in Non-Dream-Recallers (t_70_ = − 0.34, *p* = 0.73).

Interestingly, when separating participants based on if they could recall their dreams, the overnight change in emotional reactivity was only shown in Dream-Recallers. Compared to Non-Dream-Recallers, Dream-Recallers showed decreased valence (t_120_ = 2.50, *p* = 0.01, Fig. 6) and arousal (t_120_ = 2.40,* p* < 0.02, Fig. 7) ratings for negative images over a night of sleep. No difference was found for neutral images (*p*s > 0.05). The arousal and valence ratings were not significantly different (*p*s > 0.1) between the two groups during Test 1 for either negative (Dream-Recallers: M_valence_ = 5.97 ± 1.12, M_arousal_ = 4.70 ± 1.96; Non-Dream-Recallers: M_valence_ = 5.73 ± 1.92, M_arousal_ = 4.11 ± 2.33); or neutral images (Dream-Recallers: M_valence_ = 2.01 ± 0.86, M_arousal_ = 2.19 ± 0.95; Non-Dream-Recallers: M_valence_ = 2.08 ± 1.15, M_arousal_ = 2.25 ± 1.29).

**Figure 8:**
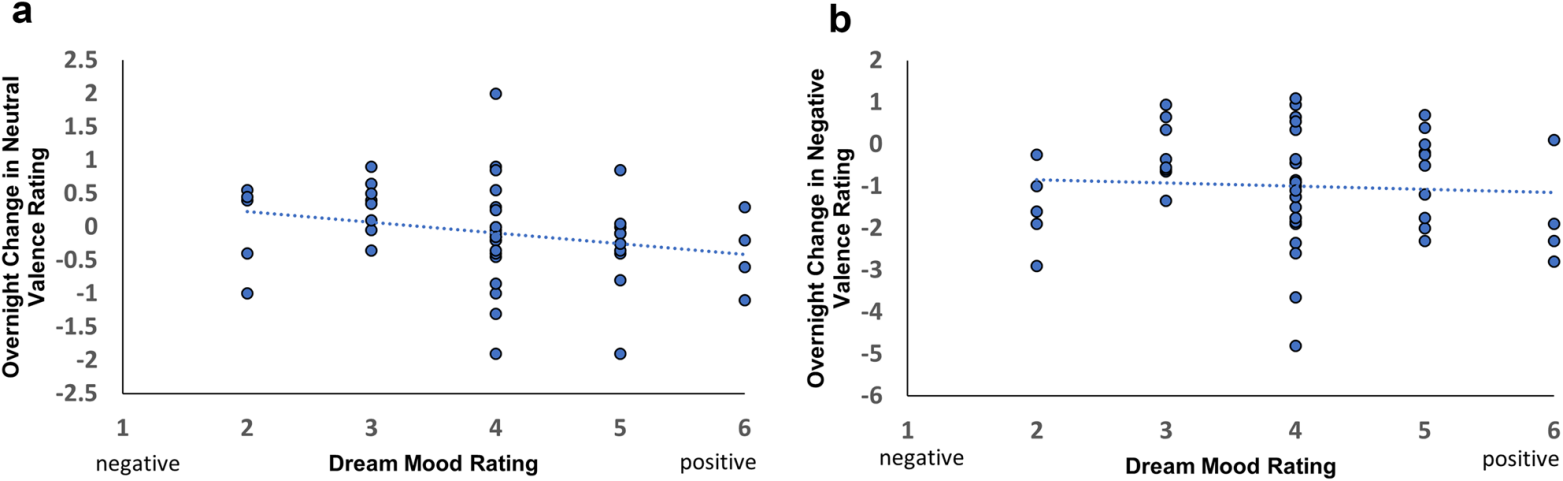
Association between dream mood and change in emotional valence. (**a**) A negative correlation between dream mood and overnight valence change for neutral pictures indicates more positive dreams are associated with more reduction in valence rating (rho = − 0.31, *p* = 0.03). (**b**) No significant correlation between dream mood and change in emotional valence was found for negative pictures (rho = − 0.08, *p* = 0.60).

Given the contrasting predictions that the mood of dreams either reflects (passive) or regulates (active) waking experience, we examined how the mood of participants’ dreams impacted their next day performance. We found a negative correlation between dream mood (higher rating is more positive) and the overnight change in valence rating for neutral images (rho = − 0.31, *p* = 0.03, Fig. 8a), but not for negative images (rho = − 0.08, *p* = 0.60, Fig. 8b). In other words, more positive dreams led to greater reduction in next-day reactivity to neutral pictures, implicating an active role of dreaming in emotional regulation. We found no correlation between dream mood and memory performance or arousal ratings (*p*s > 0.05). Dream mood was positively correlated with emotion regulation as measured by DERS (rho = 0.35, *p* = 0.02), suggesting negative dreams are associated with less difficulty in emotional regulation on a trait level.

To further discern the role of dreaming in emotional processing, we explored how dream mood ratings were correlated with valence and arousal ratings the next morning. If dream mood ratings were reflective of overall mood, we would expect a positive correlation between dream mood ratings and valence and arousal ratings the next morning. We found dream mood rating was not significantly correlated with valence (negative images: rho = − 0.09, *p* = 0.53, neutral images: rho = − 0.20, *p* = 0.17) or arousal ratings (negative images: rho = − 0.04, *p* = 0.78, neutral images: rho = − 0.17, *p* = 0.24) the next morning at Test 2, indicating a lack of continuity between the dream mood rating and overall emotional reactivity.

Another potential factor influencing the relationship between dream mood and emotional reactivity is daytime mood. It's conceivable that individuals might react more favorably to emotional stimuli if they're in a good mood during the day. We assessed participants' daytime mood from the sleep diary, but found no significant correlation between daytime mood rating and dream mood (rho = − 0.18, *p* = 0.23). There were also no significant correlations between daytime mood rating and valence and arousal ratings at Test 2 (*p*s > 0.2) or changes overnight (ps > 0.09). Additionally, the daytime mood rating didn’t differ between Dream-Recallers and Non-Dream-Recallers (t_113_ = 0.92, *p* = 0.36). These findings suggest that daytime mood rating did not influence either dream mood or emotional reactivity. However, it's possible that participants' mood upon awakening, rather than their average mood throughout the day, could impact dream mood and emotional reactivity—a metric not captured in the present study.

## Discussion

Our study highlights the critical role of dreaming in emotional memory processing during sleep. First, we replicated prior reports of an emotional memory tradeoff with sleep enhancing consolidation of memories for negative over neutral stimuli^28,54^. Remarkably, this tradeoff was only present in participants who reported dreaming during the offline sleep period, whereas no differences were found between negative and neutral memory performance in those who did not report dreaming. Again, this categorization does not distinguish whether a person actually dreamed without recollecting the dream or whether they in fact didn’t dream at all, an important topic for future research. Additionally, building on prior results that emotional reactivity to prior experiences decreases over a night of sleep, we showed that the decreased emotional reactivity was only present in Dream-Recallers. Importantly, dream mood predicted overnight decreases in reactivity, but was not correlated with next-day emotional reactivity rating or next day overall mood, suggesting participants’ rating for dreams was not directly associated with their morning emotional reactivity but with the sleep-dependent change in emotional reactivity. Together, these results are consistent with the hypothesis that dreams play an active, rather than a passive, role in emotional memory processing whereby dreaming attenuates emotional reactivity and biases offline memory consolidation towards emotionally charged content at the cost of neutral memories. Furthermore, the emotionality of dreams affects the emotion regulatory process, with more positive dream content associated with decreases in next-day ratings of valence in neutral images.

Our findings also indicate a significant role of sleep duration in emotional memory consolidation. Specifically, longer sleep was associated with greater memory for negative, but not neutral, stimuli in Non-Dream Recallers only. Additionally, we observed an effect of sleep duration on the interaction between dreaming and emotional memory consolidation. Together, these findings suggest that longer sleep time, coupled with reduced morning homeostatic sleep pressure, might not only increase the likelihood of recalling a dream, but also enhance the retention of negatively salient memories when participants were not able to recall a dream. These insights underscore the multifaceted nature of sleep and its impact on emotional processing and memory consolidation. From our data, we can conclude that extended sleep functions to enhance negative memories, but not necessarily the forgetting of neutral memories, while dream recall may specifically support the trade-off between the two. Yet, we do not understand the mechanisms by which these factors, dream recall and extended sleep, independently or synergistically contribute to this memory condition. Future studies that extend these findings with different methodologies are required to elucidate the mechanisms through which total sleep time impacts dreaming and emotional processing.

Studies on the emotional memory trade-off effect across sleep have shown mixed effects. A recent meta-analysis examined 34 sleep studies that measured memory for emotional and neutral content across a nap or a night of sleep, versus a wake period, and found no memory differences between content categories (emotional/neutral)^55^. However, similar to other meta-analyses that probe sleep’s impact on memory consolidation^56^, this null finding is due to a limited methodological approach in these papers that base their conclusions on post-sleep performance measures alone, rather than a pre-sleep to post-sleep performance difference score that accounts for baseline. This is a critical factor because episodic memories are information-specific, as such the trajectory of memory from encoding to recall is the relevant measure when examining the impact of sleep. Meta-analyses that do take into account pre-post performance differences, compared to wake, report significantly more benefits to episodic memory generally^57^ and the emotional memory trade-off effect specifically^58^. In addition, studies have also revealed a potential role of REM sleep in this process. For example, one study reported that emotionally-charged texts were more likely to be remembered after a period of REM-rich sleep compared to NREM sleep^59,60^. The amount of REM sleep was also positively correlated with emotional memory trade-off over a nap^61^, and REM sleep selectively maintained emotional over neutral memory compared to slow-wave sleep^55^. These studies all help to illustrate the connection between memory for emotional salient events and REM sleep, but they also lack mechanistic explanation.

Neural mechanisms supporting emotional memory consolidation have been proposed. Specifically, emotional experiences are more likely to be tagged with stress and arousal related neurotransmitters, such as cortisol and norepinephrine, during wakefulness, signaling greater significance and prioritization for further processing and consolidation during sleep^62,63^. The memory component may be strengthened via spindle-mediated reactivation processes during NREM^64^, followed by further reactivation during REM sleep via amygdala activation^65–71^. Building on these findings, the affect tagging and consolidation (ATaC) model posits that negative experiences that heighten amygdala activity during waking are preferentially reactivated and processed during REM sleep to create a biased consolidation of negative memories^72^. Several prominent dream theorists have posited that the amygdala plays a central role in generating, modulating, and recalling emotional dream experiences, particularly those characterized by negative emotions^21,73,74^. Dreaming may reflect heightened amygdala activity and may be the mechanism by which tagged memories are afforded extra processing by REM sleep when dreams are most vivid^75^. However, this is not directly supported in our study as we did not know which sleep stage dreams occurred due to lack of EEG data.

Our results also show that dreaming is associated with attenuated reactivity to emotional stimuli, which shares similarities to prior studies. For example, Hutchinson showed decreased arousal to negative stimuli when reactivating memories during REM sleep, but not slow wave sleep (SWS)^76^. In this study, participants rated arousal to negative and neutral picture-sound pairs before and after sleep. During sleep, researchers replayed half of the associated sounds during REM or SWS. They found that the arousal rating to negative stimuli decreased significantly when replayed during REM. Furthermore, an overnight decrease in amygdala reactivity to emotional stimuli has been found to be associated with the duration of REM sleep^67,77^. This decrease in reactivity was also linked to increased connectivity between the amygdala and prefrontal cortex, and correlated with decreased REM sleep gamma frequency—a proxy for adrenergic activity^34^. Notably, van der Helm and colleagues proposed that the marked suppression of central adrenergic neurotransmitters during REM sleep, commonly implicated in arousal and stress, is coupled with activation in amygdala-hippocampal networks responsible for encoding salient events while attenuating emotional reactivity. They proposed the sleep to forget and sleep to remember (SFSR) model, which states that REM sleep facilitates the consolidation of emotional memories while downscaling emotional reactivity^32^. Our results build on the SFSR model by implicating dreaming as the context for emotional memory processing. However, one study reported participants with the lowest intensities of negative emotions in dreams showed the highest morning decreases in negativity ratings, which is inconsistent with our findings^38^. This could be due to methodology difference. They used a serial-awakening paradigm where participants were awakened from REM sleep multiple times throughout the night, whereas we collected one dream report in the morning. One possible alternative explanation for our results is that the salient dream memory that participants asked to recall in the morning might have interfered with the memory of the less salient neutral pictures. Future studies should address the role of dreaming in the emotional memory trade-off using a wide range of techniques of probe dreaming and dream content.

The emotional memory trade-off necessarily involves the forgetting of neutral information in exchange for retention of negative memories. Similarly, the overnight reduction in emotional reactivity can also be interpreted as forgetting (i.e., reduced response) at the level of emotional response. Conceived in this way, our results show forgetting in both the memory and the emotion reactivity aspects, suggesting that one potential way that dreaming plays a role in prioritizing consolidation of emotionally salient memories is by facilitating successful forgetting. In line with this hypothesis, studies have shown that REM sleep was associated with forgetting of details while generalizing memories to novel situations^78,79^. These studies suggest that the neural dynamics during REM sleep create an optimal environment for forgetting, which allows for the elimination of irrelevant information to make space for new memories. On the neuronal level, the locus coeruleus is relatively inactive during REM sleep, which leads to decreased noradrenergic activity and allows for a decrease in synaptic efficiency^80–83^. In other words, the decreased noradrenergic activity during REM sleep provides synaptic circuits with an opportunity to reset. On the synaptic level, Zhou and colleagues reported that REM sleep facilitates experience-dependent dendritic elimination, a process implicated in forgetting^84^. Dendritic elimination is the selective elimination of weak connections between neurons, which is crucial for neural plasticity, the brain's ability to adapt and change in response to new experiences^85^. REM sleep deprivation led to decreased dendritic elimination and impaired learning in fear conditioning and monocular deprivation^86^. Furthermore, blockade of dendritic calcium spikes with an NMDA receptor antagonist during REM sleep led to a decrease in dendritic spine elimination, suggesting the significant involvement of calcium spikes during REM sleep in the promotion of experience-dependent dendritic spine elimination and forgetting^86^. REM sleep also facilitates forgetting via melanin-concentrating hormone (MCH) neurons^87^. In this study, inhibiting MCH neurons during REM sleep improved memory, an effect not seen in wake, suggesting REM-active MCH neurons facilitates forgetting. REM-guided decreased noradrenergic activity, synaptic elimination, and MCH cell induced forgetting may be a candidate for the neural mechanisms of dream-related emotional memory trade-off and attenuated emotional reactivity.

The current study has several limitations. We used a commercial wearable device to monitor sleep activity, which only provides a crude picture of the sleep architecture and doesn’t allow for detailed analyses into brain activity. Only one dream report upon awakening in the morning was collected for each participant, which limits our ability to differentiate NREM and REM dreams, which might have different roles in memory^88^. While new methodologies for assessing dreaming in humans continue to evolve, two primary methods currently dominate the field: mid-sleep and post-sleep dream reports. Our study engaged the post-sleep dream report method due to its technical simplicity, which suited our mixed-setting design (i.e., in-lab and remote). We also used this method because morning reports occur during a REM-rich sleep period, a brain state previously associated with the selective processing of emotional experiences^67^. As such, morning reports may offer the advantage of engaging more emotional experiences — a hypothesis we intend to explore further in future experimental designs utilizing mid-sleep dream reports. Although we accounted for individual difference in depression and anxiety, there are other factors that influence the emotionality of dreams, such as sleep quality^89^ and PTSD^90^. Morning mood might mediate the association between dream mood and emotional reactivity, such that when people wake up in a positive mood, they rate their dream more positively and can better regulate their emotions towards negative stimuli. Although our current study did not reveal any impact of daytime mood on dream mood or emotional reactivity, we did not specifically investigate the influence of morning mood. Future studies are needed to explore the role of morning mood in this process as it was not collected in this study. Moreover, sex hormones affect REM sleep theta power and locus coeruleus activity, both of which are critical to emotional processing^91^. Our study did not account for the fluctuations in sex hormone levels among our participants, which could be a focus for future studies.

Taken together, our findings suggest that dreaming actively engages in the complex processing of emotions, preserving emotionally salient memories while simultaneously facilitating the forgetting of irrelevant information and the attenuation of emotional reactivity. This intricate phenomenon likely involves a network of neural mechanisms operating during REM sleep that promote the reactivation of amygdala-related neural circuits, the modulation of adrenergic activity, and the interplay between stress and arousal-related neurotransmitters. REM sleep also facilitates forgetting via decreased noradrenergic activity and dendritic elimination. Dreaming, intimately intertwined with memory and emotional processing, may serve as a context for the successful consolidation and integration of emotionally salient experiences, as well as the forgetting of non-salient memories and emotions. Dreams act as a dynamic stage for the processing and integration of emotional content, facilitating emotional regulation and the resolution of emotional conflicts. These insights significantly advance our understanding of the intricate function of dreaming, a longstanding question that has puzzled scientists, philosophers, and spiritualists for centuries. By unraveling the complex interplay between dreaming and emotional processing, our research sheds new light on the fundamental role of sleep in emotional well-being and adaptive cognitive processes.

## Data Availability

The datasets generated during and/or analyzed during the current study will be available from the corresponding author on reasonable request.
